# Nanomaterials as Photocatalysts—Synthesis and Their Potential Applications

**DOI:** 10.3390/ma16010193

**Published:** 2022-12-25

**Authors:** Agnieszka Feliczak-Guzik

**Affiliations:** Faculty of Chemistry, Adam Mickiewicz University in Poznań, Uniwersytetu Poznańskiego 8, 61-614 Poznań, Poland; agaguzik@amu.edu.pl

**Keywords:** nanomaterials, semiconductors, photocatalyst, photocatalysis, plasmonic properties of metals

## Abstract

Increasing demand for energy and environmental degradation are the most serious problems facing the man. An interesting issue that can contribute to solving these problems is the use of photocatalysis. According to literature, solar energy in the presence of a photocatalyst can effectively (i) be converted into electricity/fuel, (ii) break down chemical and microbial pollutants, and (iii) help water purification. Therefore, the search for new, efficient, and stable photocatalysts with high application potential is a point of great interest. The photocatalysts must be characterized by the ability to absorb radiation from a wide spectral range of light, the appropriate position of the semiconductor energy bands in relation to the redox reaction potentials, and the long diffusion path of charge carriers, besides the thermodynamic, electrochemical, and photoelectrochemical stabilities. Meeting these requirements by semiconductors is very difficult. Therefore, efforts are being made to increase the efficiency of photo processes by changing the electron structure, surface morphology, and crystal structure of semiconductors. This paper reviews the recent literature covering the synthesis and application of nanomaterials in photocatalysis.

## 1. Heterogeneous Photocatalysis

According to the International Union of Pure and Applied Chemistry, heterogeneous photocatalysis is a reaction in which a photocatalyst initiates the process after absorption of the exciting radiation and the photocatalyst occurs in a different thermodynamic phase than the reactants [1,2]. Among the materials most commonly used as photocatalysts are solid semiconductors, primarily transition metal oxides. The specific resistance of semiconductors at room temperature ranges from 10^−2^ Ωcm to 10^9^ Ωcm and is strongly temperature dependent. Due to their conductive properties, semiconductors are intermediate between dielectrics (insulators) and conductors (metals) [2]. In semiconductor materials, the valence band (VB) is fully occupied, while the conduction band (CB) is completely empty at absolute zero temperature. The energy of the excited band (Eg) in such materials is in the range of 0–4 eV, and the Fermi level lies between the conduction band and the valence band. For semiconductors the forbidden band in a macroscopic scale assumes constant values. 

Heterogeneous photocatalysis includes a wide range of chemical reactions, for example: partial or complete oxidation, dehydrogenation, hydrogen transfer, oxygen and deuterium isotopic exchange, metal deposition, water detoxification, and removal of gaseous pollutants [2]. 

**Figure 1:**
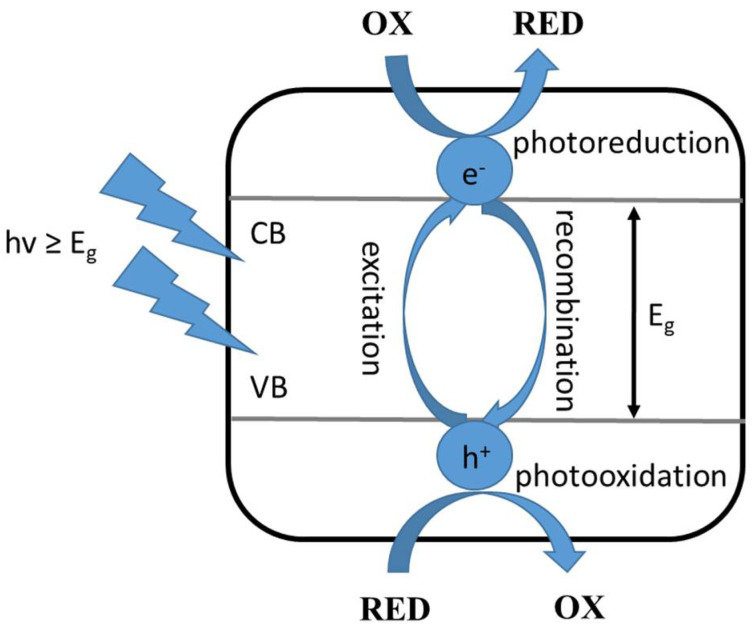
Excitation of a semiconductor photocatalyst, where: OX—oxidized compound, RED—reduced compound, VB—valence band, CB—conduction band, Eg—the energy of the excited band, hv—the energy of the radiation quantum [3].

This type of photocatalysis involves the following steps: (i) adsorption of the substrates involved in the reaction on the surface of the photocatalyst, (ii) absorption of radiation quanta of appropriate energy by the applied photocatalyst, (iii) generation of reactive electron-hole pairs, and (iv) electron and hole reactions with the adsorbed compounds or their recombination. To begin with, excitation of the photocatalyst, which is a semiconductor, involves absorption of radiation with an energy equal to, or greater than, the energy gap, followed by excitation of an electron from the valence band to the conduction band. Then, the resulting individuals react with the surrounding components: the electron causes reduction (photoreduction) and the hole causes oxidation (photooxidation) of the compounds adsorbed on the applied photocatalyst, as shown in Figure 1 [3]. 

Absorption of light by a semiconductor generates the formation of an electron-hole pair, increasing the concentrations of electrons and holes above their equilibrium concentration by Δn* and Δp*, respectively. This occurs until the recombination reaction slows down the process. The free energy of these individual charge carriers is expressed by the so-called quasi-Fermi level [4]. For the minority charges, the position of the quasi-Fermi level differs significantly from that of the Fermi level of an unlit semiconductor, while the difference is small for the majority charges. 

It should be added here that the absorption of light depends primarily on the distance from the surface of the semiconductor. This means that the distribution of excess charges is not uniform. As the distance from the semiconductor surface increases, the probability of a minority charge appearance decreases. However, the biggest changes will occur near the surface itself, where the minority charges accumulate. The change in charge density has a major impact on all processes occurring in this area [4].

On the basis of the compositions of the initial materials, Yang et al. grouped photocatalysts into six categories: (i) traditional semiconductor, (ii) molecular, (iii) plasmonic, (iv) 2D, (v) quantum dots, and (vi) traditional semiconductor-based photovoltaic assisted [5]. Of the aforementioned, plasmonic nanostructures, which can be used to enhance light absorption by semiconductors or to drive direct photocatalysis with visible light on their surface, are currently attracting considerable attention. Interest in this type of materials, may also be due to the fact that photocatalytic processes carried out using semiconductors show some limitations, such as: low absorption coefficient, limited wavelength range for light, and low selectivity towards a specific chemical reaction pathway. 

Plasmonic nanostructures can confine electromagnetic energy in free space to nanometer-sized regions and convert it into various forms, including confined and scattering fields, high-energy "hot" electrons, and holes, or heat and thermal radiation. These nanostructures are designed in principle to mainly express one of such energy transformations; their properties depend on where the nanostructures are to be used [6].

The Localized Surface Plasmon Resonance (LSPR) generated in plasmonic structures can lead to enhancement or formation of linear and nonlinear optics phenomena (e.g., spontaneous emission, nonlinear absorption or Raman scattering) [7]. LSPR occurs when an electromagnetic wave with a frequency identical to the vibrational frequency of localized surface plasmons falls on a plasmonic nanoparticle [8]. The main feature of the localized resonance of surface plasmons is that its frequency can be varied by selecting both the size, shape, and position of the nanostructures, as well as the type of matrix and material of which they are made. This makes it possible to control the resonance and adjust it to those wavelengths to be used in the planned applications. 

## 2. The Properties of Photocatalysts

Photocatalysts, compared to traditional catalysts, operate on a different principle. 

Table 1 compares the properties of traditional catalysts with those of photocatalysts [9].

**Figure 2:**
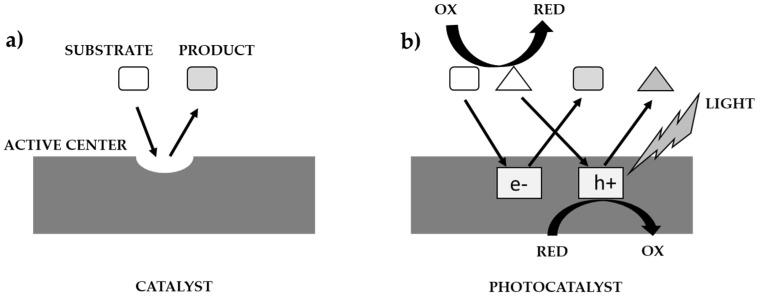
Comparison of catalysis process (**a**) with photocatalysis (**b**) [10].

The course of the catalytic, and photocatalytic processes, according to Ohtanie using a catalyst, and a photocatalyst is shown in Figure 2 [10]. Most researchers use the term ’photocatalytic activity’, but in almost all cases the meaning is the same as absolute or relative reaction rate. The reason for this may be to get others to think of ’photocatalytic reaction rate’ as one of the properties of a photocatalyst (i.e., photocatalysts have an individual activity, whereas “reaction rate” is controlled by activity under given reaction conditions). In catalysis, “catalytic activity” (Figure 2a) was used to show the properties (or performance of the catalyst) because the ‘active site’ on the catalyst is responsible for the catalytic reaction. The reaction rate per active site can be estimated and should be the “catalytic activity”. On the other hand, there are no active sites on the photocatalyst, in the same sense as in thermal catalysis, i.e., the rate of the catalytic reaction is predominantly governed by the number of active sites, and the reaction rate strongly depends on various factors, e.g.: the intensity of the irradiated light that initiates the photocatalytic reaction (Figure 2b). Given that the dark side of a photocatalyst or suspension has no effect on the photocatalytic reaction, the use of the term ‘active site’ is inappropriate, and therefore a relationship between photocatalytic activity and active sites cannot be expected. In the kinetic analysis of general chemical reactions, the rate constant is estimated and compared. Given that photoexcited electrons (e^−^) and positive holes (h^+^) induce a redox reaction, the rate constant of these active species can be estimated. Since e- and h+ recombine with each other, the overall rate of the photocatalytic reaction also depends on this recombination rate. Assuming that k(redox) and k(recombination) are the rate constants of the reaction rates occurring by e^−^ and h^+^ and their recombination, respectively, i.e., in the simplest kinetic model, the ratio k(redox)/k(recombination) should be a measure of the intrinsic photocatalytic activity [10].

Great influence on the properties of a given semiconducting material applicable in photocatalysis has its size. As the size of the crystal is minimized, the ratio of the number of atoms on its surface to those inside the crystal increases, which causes a change not only in the surface properties of the semiconductor but also in the entire material. It affects, among other things: the melting point of the material and the electron properties - if the size of the semiconductor decreases to a few nanometers, separate energy levels are created instead of continuous energy bands (this is called the quantum size effect) [11].

In nanoparticles, the electron and hole are closer to each other than in bulk semiconductors, which has the effect of increasing the Coulombic interactions between them. This also affects the size of the energy gap. Increasing the energy gap allows the absorption threshold to shift toward shorter wavelengths as the particle size decreases. Consequently, this leads to an increase in the molar absorption coefficient, which is related to the overlap of the wave functions of the charge carriers. This has become the basis for the use of nanomaterials (nano semiconductors) in catalysis, luminescence, optoelectronic devices, and solar cells [12].

## 3. Synthesis of Photocatalysts

Over the past few years, the synthesis of nanomaterials has been a dominant trend in many fields of science and technology [13]. Nanomaterials of metallic or semiconducting nature that can be excited by radiation in the UV-Vis range have become very popular [3]. These materials differ in characteristics from micrometric-sized materials because nanometer-sized particles exhibit new and unique magnetic, electrical, optical, and catalytic properties [3]. A good photocatalyst should be characterized by: (i) the ability to absorb radiation from a wide spectral range of light, (ii) the appropriate position of the energy bands of the semiconductor about the redox reaction potentials, (iii) high mobility and long diffusion path of charge carriers, (iv) thermodynamic, electrochemical, and photoelectrochemical stability [14]. Moreover, for the reactions involving the resulting photocarriers to occur efficiently, it is necessary to effectively prevent recombination by separating electron-hole pairs, and then their transport to the semiconductor surface. Meeting these requirements by semiconductors is very difficult. Therefore, efforts are being made to increase the efficiency of photo processes by changing the electron structure, surface morphology, and crystal structure of semiconductors [10]. Broadening the range of radiation absorption can be achieved by, among other things, doping the cationic and/or anionic subgrid, or introducing plasmonic metal nanoparticles. An increase in the degree of crystallinity of the resulting materials by reducing the concentration of defects leads to a reduction in the number of recombination centers. Reducing the size of particles, while increasing the specific surface area, has a beneficial effect on the efficient diffusion of charge carriers to the surface. The selection of the shape of nanocrystals by adjusting the proportion of selected crystallographic planes differing in surface energy allows the adsorption of only selected particles, thus providing selectivity of photocatalytic processes [10]. 

### 3.1. Synthesis of Semiconductors

The optical and electrical properties of semiconducting materials are strongly related to the distribution of energy bands, or more precisely, to the energy of the excited band. It determines the threshold energy that electrons must have at the moment of transition from the valence band to the conduction band. Semiconducting nanocrystals can be considered as a multi-atomic molecule in which the electron orbitals are formed: the highest occupied molecular orbital (HOMO) and the lowest unoccupied molecular orbital (LUMO). In the ground (non-excited) state, the HOMO orbital is filled, while the LUMO is unfilled. Upon excitation, electrons in the semiconducting materials can switch from HOMO to LUMO. This transition is analogous to the transitions of electrons from the highest occupied molecular orbital to the lowest unoccupied molecular orbital of organic compounds. When electrons are excited from the valence band to the conduction band, a gap of positive charge, called an "electron hole," remains in the valence band. At a later stage, as a result of recombination of electrons and holes, the energy released in this process is emitted in the form of a photon (radiative recombination) or is transferred to another charge carrier during non-radiative recombination [15].

According to the number of charge carriers formed as a result of excitation, we can distinguish between intrinsic semiconductors (the concentration of free electrons of a semiconductor is equal to the concentration of holes) and doped semiconductors, in which the introduction of a doping material generates carriers of one type. Hence, we distinguish between n-type (over-doped) semiconductors, in which there is electron overshoot, and p-type (undoped) semiconductors, which are characterized by hole overshoot (the number of holes is greater than the number of electrons in the conduction band). Intrinsic semiconductors include materials made of a single type of atoms, such as B, Ge, Si, Se, S, Sb, Te, or I. Germanium, silicon, and selenium are known as intrinsic semiconductors, while the other elements are most often used as dopants or as components of the so-called complex (non-self-contained semiconductors) semiconductor mate-materials. The group of compound semiconductor materials includes chemical compounds of two, three, or more chemical elements, of which the most common are semiconductors of two-element compounds of the type A(III)B(V), A(II)B(V), A(II)B(VI), or A(IV)B(VI). Depending on the elements that make up the semiconductors in question, they can exhibit both p-type (ZnTe) and n-type (ZnSe) conductivity. A(II)B(VI) type semiconductors, which include ZnS, ZnSe, ZnTe, CdS, CdTe, HgSe, HgTe, and HgS, are used as luminophores in the visible light range for the production of optical fibers and photovoltaic components [16]. Depending on the type of absorption occurring in semiconductor materials, a distinction is made between the semiconductors with a straight or oblique interband gap. For the semiconductors with a straight gap, the bottom of the conduction band and the top of the valence band occur for the same value of k (lattice vector), which determines the position of the unfilled state in the band. For the semiconductors with a straight transition for effective absorption of light there must be a probability of meeting of two particles—an electron and a photon. In oblique transitions, three particles—an electron, a photon, and a phonon—should meet. This means that the absorption coefficient for straight transitions takes a higher value than for oblique transitions [17]. Therefore, semiconductors with straight transitions are used as luminophores; they have high luminescence efficiency [18].

#### 3.1.1. Nanoscale Semiconductors

At the nanoscale, the physicochemical properties of materials change in a fundamental way compared to those of their bulk counterparts [15], which is related to the so-called quantum entrapment effect [19]. It occurs as the particle size decreases below the Bohr radius of the exciton, causing the electron in the nanocrystal to behave as if in a three-dimensional box of potential. As the Bohr radius of the exciton increases, the energy of the excited band decreases [20]. The quantum confinement effect plays a key role in the properties of nanocrystals, which is related to the change in the energy of the excited band. In semiconductor nanocrystals, analogous to organic molecules, a photon can be released or absorbed at the transition of charge carriers between the quantum levels of the valence band and the conduction band. The wavelength of absorption or luminescence can be controlled by changing the size of the quantum dots, which are often called “artificial atoms” [21].

Doping semiconductor nanocrystals affects their electrical, optical or magnetic properties. It may cause an increase in conductivity (an increase in the number of electrons or holes), the formation of a new energy level, which in turn contributes to the appearance of completely new optoelectronic properties of nanocrystals [22]. In A(II)B(VI) type semiconductors, doping with atoms of other elements has the greatest impact on their luminescent properties. For example, CdS quantum dots (4.2 nm) doped with Mn^2+^ ions show a blue shift in photoluminescence spectra and exhibit orange photoluminescence, compared to the undoped CdS nanocrystals. This phenomenon is caused by the additional 6A1-4T1 transition of Mn^2+^ ions, resulting in an increase in the quantum yield of luminescence reaching up to 41%. The observed properties of doped nanocrystals compared to undoped ones occur due to a shift in the energy levels in the nanocrystals caused by the introduction of the dopant [23,24]. Depending on the desired characteristic of the nanomaterials, selected dopants are introduced. Thus, doping zinc selenide nanocrystals with silver atoms causes a shift of the emission maximum from the blue light region to the green light region [25], and doping ZnSe NCs with Cr^2+^ atoms into the green light region, and contribute to changing the electrical properties of the nanocrystals themselves, which has been successfully applied in the production of lasers [26]. The combination of the relative ease of fabrication of semiconductor nanomaterials with the ability to adjust the position and magnitude of their bandgap energy has produced promising materials with a wide range of applications, including optoelectronics, photonics, catalysis, photovoltaics, various sensors, and biomedicine [27,28,29].

#### 3.1.2. Titanium(IV) oxide (TiO_2_)

Titanium(IV) oxide is a semiconductor material with high efficiency in various photocatalytic reactions. It exhibits high chemical and photochemical stability. 

However, besides the above-mentioned advantages, has some limitations, viz:

- the possibility of its wide application is limited by, e.g., the fact that it absorbs only UV radiation (387.5 nm), which needs the use of the light sources of high cost of exploitation [30];
- a decrease in the efficiency of the photocatalytic reaction, which is related to the phenomenon of recombination of photo-excited charge carriers (electrons (e^−^) and holes (h^+^)) [31];
- low selectivity especially in photo-oxidation reactions of organic compounds [32].

In view of above-mentioned limitations, work has begun on the synthesis of new photocatalysts on titanium(IV) oxide matrix, whose photocatalytic activity would be in the visible radiation range (>400 nm). This would significantly expand the applicability of heterogeneous photocatalysis in environmental protection, either by using the main part of the sunlight spectrum or by using a light source with lower irradiance. Currently, the goal of most of the work carried out in the world is to obtain a visible-light-activated photocatalyst, which would be obtained by modification of TiO_2_. Reactions carried out under hydrothermal conditions made it possible to obtain semiconductor materials with different morphological structures, for example, during the synthesis of anatase (TiO_2_), it was important to use appropriate substances to control the formation of crystal morphology during hydrothermal synthesis, including fluorine compounds [33]. However, these compounds at higher temperatures can undergo transformations to highly toxic compounds with corrosive properties. This poses quite a limitation to the applications of this method [34]. An alternative to hydrothermal methods, for the synthesis of anatase, can be the process of crystallization in the gas phase. In this process, it is possible to obtain anatase crystals with a decahedral structure (decahedral anatase particles) [34]. Amano and colleagues showed that rapid heating, up to 1200 °C, and cooling of a mixture of titanium(IV) chloride and oxygen promoted the formation of anatase in the form of decahedral-shaped crystals [35]. However, this method did not permit for controlled and continuous dosing of titanium(IV) chloride (TiCl_4_) into the reactor system, which was connected to a coaxial flow of reaction gases. This was a key element in the controlled preparation of anatase crystals with different morphological parameters. Hence, Janczarek and co-workers [36] have developed a method for precise dosing of titanium(IV) chloride vapor into the tubular reactor space, combined with a constant flow of oxygen. This solution made it possible to obtain a product characterized by well-defined properties with high efficiency and reproducibility.

The efficiency of heterogeneous photocatalysis using TiO_2_ as a photocatalyst depends primarily on the polymorphic variety of the material. Polymorphic varieties of titanium oxide include rutile, anatase, and brucite [37]. The most desirable form of TiO_2_ is anatase, which is characterized by a large specific surface area, a high degree of surface hydroxylation, and a bandgap energy of Eg = 3.23 eV (384 nm). Rutile (Eg = 3.02 eV (411 nm)) is less effective in photocatalytic processes, which is due to the presence of differences in the recombination rates of electron-hole pairs; the recombination time between the electron (e^−^) and the hole (h^+^) for rutile is shorter than their migration time to the surface. Besides, rutile has fewer active sites and hydroxyl groups on the surface compared to anatase [38].

TiO_2_-based materials with enhanced UV activity or activity under visible light can also be obtained by: (i) the addition of transition metal ions, e.g., Mn, Nb, V, Fe, Au, Ag [39,40]; (ii) preparation of a reduced form of TiO_2_ [41]; (iii) sensitization of TiO_2_ with dyes [42] and with semiconductors with a smaller Eg bandwidth [43], (iv) doping with non-metals, e.g.: nitrogen [44], carbon [45], or phosphorus [46]. 

The mechanism of excitation of the photocatalyst depends on how the material is modified. The main types of excitation of TiO_2_ under the influence of radiation from the visible range include [41,47,48]: (a) the appearance of a new energy state associated with the presence of an oxygen vacancy, (b) dye sensitization, where the dye is a sensitizer, (c) dye sensitization, where the dye is both a sensitizer and a degradant, and (d) the formation of a new energy level below the conduction band associated with the presence of metal cations. 

Another method of titanium(IV) oxide modification is its doping with metals or non-metals, such as boron, tungsten, or precious metals. The doping with boron can enhance the photocatalytic activity of titanium(IV) oxide under visible light. 

The introduction of boron into the TiO_2_ structure inhibits the growth of crystal size, can affect the phase transformation of anatase to rutile, and can increase the specific surface area of photocatalysts [49,50,51,52,53].

Tungsten oxide, on the other hand, due to the width of its excited band (2.8 eV), can be used as an admixture of titanium dioxide, thus causing an increase in its photocatalytic activity in the visible light range [54,55,56,57,58]. 

Modification with noble metals (primarily gold, silver, or platinum) can enhance the activity of titanium dioxide in the visible light range since nanoparticles of noble metals such as silver and gold exhibit the ability to absorb visible radiation, which is a result of the existence of a surface plasmon. This enables them to absorb light in the visible and near-infrared range, which favors their potential use for activating titanium dioxide with solar radiation. In addition, they can capture charge carriers (e^−^/h^+^), and thus cause a reduction in the rate of the recombination process of electron-hole pairs, which is associated with an increase in the quantum yield of the reaction [59]. 

It has been observed that the photocatalytic activity of TiO_2_ modified with noble metals depends, among other things, on the size of the metal particles. The size of the obtained noble metal nanoparticles is affected by the reaction temperature, the reducing reagents used, the type of stabilizer used, and other factors [60,61,62,63]. 

#### 3.1.3. Zinc Oxide (ZnO)

One of the commonly used photocatalysts, along with TiO_2_, is zinc oxide ZnO. It is a material of increasing interest due to its ability to form various nanostructures such as nanowires, nanobelts, nanoscratches, nanospheres, nanofibers, and nanotetrapods. Currently, however, nanowires of zinc oxide are of the greatest interest, especially when arranged in layers oriented in perpendicular to the conducting substrate. Nanowires deposited in this way are characterized by a high diameter-to-height ratio, which means that the total surface area of the deposited ZnO can be up to 100 times greater than the geometric surface area on which this deposition occurs. Consequently, a large amount of photosensitive material can be deposited on the ZnO surface, resulting in a high light absorption efficiency value. The ordered nanowire layers are used, for example, in lasers, electroluminescent devices, sensors, photocatalytic systems, and third-generation solar cells [64,65]. 

In the synthesis of ZnO, a key process is the preparation of zinc hydroxide. There are several natural forms of Zn(OH)_2_, denoted as: α-, β-, γ-, δ-, ε- Zn(OH)_2_. The latter is the most stable. Usually, during deposition, the α- form is deposited first, which under aging changes to the ϵ- form [66].

The main crystalline form of zinc oxide is wurtzite, the form that is thermodynamically stable under normal atmospheric conditions. It is a system consisting of 4O^2–^ and Zn^2+^ ions arranged in a characteristic manner. A characteristic feature of ZnO is the presence of polar and non-polar crystal planes [67].

#### 3.1.4. Comparison of the Properties of TiO_2_ and ZnO

Despite the promising properties of zinc oxide, titanium(IV) oxide is still the most commonly used photocatalyst. This is largely related to the higher chemical stability of TiO_2_. Titanium(IV) oxide has a similar energy gap to that of ZnO (3.2 eV) and a similar energy band pattern. In addition, TiO_2_ also has the advantage of higher electrical permeability than ZnO, which allows it to better retain electrons and inhibit the recombination process [68]. An advantageous feature of ZnO over TiO_2_ is also that it is a straight energy gap semiconductor (unlike TiO_2_, whose energy gap type depends on the crystallographic form). In addition, ZnO exhibits a higher electron mobility than TiO_2_ (200 cm^2^ /Vs for ZnO and 10 cm^2^ /Vs for TiO_2_) [69]. This results in a lower resistance of ZnO. The ease of fabrication and the low cost of the process may also be in favor of ZnO over TiO_2_.

### 3.2. Plasmonic Materials

Plasmonics is concerned with the studies of plasmons that are the quasiparticles made of quanta of plasma oscillations at the characteristic plasma frequency ω_p_, as a result of the action of an electromagnetic wave on quasi-free carriers originating from the conduction band of a metal or semiconductor [70]. As a result of the electromagnetic field, the quasi-submissive carriers move away from the positively charged atomic nucleus and then return to their previous state when the field no longer acts, due to the attractive Coulombic forces [71]. 

The two main groups of methods for obtaining plasmonic materials include the so-called top-down methods (building from the top down) and bottom-up methods (building the material from scratch, atom by atom, or particle by particle) [72,73]. 

Top-down methods include lithographic techniques, which include lithographic nano-printing, soft lithographic methods, or methods based on the use of a scanning tunneling microscope (STM) and 3D Direct Laser Writing [72,73]. 

#### 3.2.1. Lithographic Techniques

Electron Beam Lithography (EBL) and Focus Ion Beam (FIB) lithography favor obtaining the desired nanostructure in two steps, i.e.: hardening the resist with an electron or ion beam, and etching the nanostructure by deep plasma etching, e.g., using the Reactive Ion Etching (RIE) technique. The resulting nanostructures have very high resolution (on the order of a few nanometers). The photolithography technique, on the other hand, is based on the use of a light beam, in which a specially prepared mask—a metallic plate with appropriately selected holes through which the light beam is passed—is additionally used to obtain the desired nanostructure [74]. 

In addition to the above-mentioned lithographic techniques, soft lithography techniques are also used, including Nanoimprint Lithography (NIL) and Room Temperature Nanoimprint Lithography (RTNIL). Using room-temperature lithographic nanoimprinting, among other things, the optically active, planar, chiral photonic metamaterials are obtained [75]. The technique is based on duplicating a nanostructure on a polymer stamp, which is formed by pouring a polymeric material onto a suitably prepared template. The template (usually a quartz substrate) is obtained by micromachining or modern lithography techniques. 

#### 3.2.2. Techniques Based on Scanning Tunneling Microscopes 

Plasmonic nanostructures can also be obtained using scanning tunneling microscopes. They control the conditions for layer growth. Obtaining a given structure is made possible by a needle that mimics the given structure while scanning the electrically conductive material [76]. 

Bottom-up methods include those using self-assembly, direct nanoparticle doping methods, and gas-phase deposition techniques [77].

#### 3.2.3. Nanoparticle Direct Doping (NPDD) 

The use of nanoparticle direct doping permits obtaining desired materials through a chemical process. The advantage of this method is the deagglomerated state of nanoparticles [77], which is vital because agglomerates of plasmonic nanoparticles are useless in plasmonics, as they exhibit no or weak resonance phenomena. The main advantages of this method, in addition to those mentioned above, is the speed of obtaining composites, the preservation of the original form of the dopant in the composite by controlling the size and shape of the introduced nanoparticles, and the possibility of obtaining composites doped with particles, both metallic and non-metallic [77]. 

An example of the materials obtained by this method is precast glass doped with silver nanoparticles (0.15 wt%) [77].

#### 3.2.4. Techniques Based on Self-Assembly

Self-organization techniques employ the mechanisms of natural self-assembly, e.g., the self-assembly of block copolymers. These techniques permit obtaining periodic domain nanostructures by microscopic phase separation [75]. The advantage of self-assembly is that it provides ordered structures with different morphologies [78]. 

On the other hand, the method of self-organization to single layers of nanoparticles involves dynamic evaporation of nanoparticles on the surface of liquid-air separation. This leads to the nucleation of islands of nanoparticles, followed by the growth of a monolayer [79]. Volume plasmonic materials can be obtained by the self-organization of liquid crystals, which yields a three-dimensional synthetic material that exhibits strong resonances in the visible range [76]. 

#### 3.2.5. Gas-Phase Deposition Techniques

There are two main varieties of gas-phase deposition: Physical Vapor Deposition (PVD) and Chemical Vapor Deposition (CVD). In the PVD methods (the so-called “clean technology” as no harmful chemicals are required), the applied coating exhibits an adhesive nature, and its properties depend on the purity of the substrate. The process involves obtaining vapors of the material, which are then transported to the surface where they condense, and the coating grows. With this method, only flat or simple shapes can be coated as the process requires rotation of the coated parts. The CVD methods, on the other hand, involve the introduction of gas substrates into a chamber, where the appropriate chemical reactions take place on the substrate, leading to the formation of a coating. With this method, it is also possible to coat three-dimensional parts, as the process does not require rotation of the workpieces. However, when using this method, there may be a problem with the difficulty of balancing the compound decomposition reactions throughout the volume of the working chamber, which is associated with the formation of less pure layers [74]. 

Among the plasmonic materials currently used, silver and gold predominate. Silver has the lowest losses for the visible and near-infrared light range [80], which permitted the use of this plasmonic material to obtain super-lenses and hyper senses [81], or to increase the efficiency of solar panels made of layers of amorphous silicon [82]. Gold, compared to silver, exhibits higher chemical stability under natural conditions, which permits the use of gold layers in plasmonic biosensors [83]. 

The materials alternative to expensive noble metals may be plasmonic materials belonging to the nitride group, e.g., zirconium nitride ZrN, titanium nitride TiN, hafnium nitride HfN and tantalum nitride TaN [84]; doped transparent conducting oxides such as aluminum-doped zinc oxide Al: ZnO, zinc oxide doped with gallium Ga:ZnO, tin oxide doped with antimony oxide Sb_2_O_3_:SnO_2_ and indium oxide doped with tin Sn:In_2_O_3_ [85], graphene [86], and the metals copper, aluminum, chromium, or iridium [87].

#### 3.2.6. Plasmonic Properties of Metals

Surface plasmons in metals have many fascinating properties, which enables their applications in optics, sensorics, photonics, and nonlinear fields. Recently, the plasmonic properties of some metals (e.g., Au, Ag, Cu, Al, Mg, Pt and Rh) have been widely studied both experimentally and theoretically, which is related to the fact that for the development of efficient synthesis of nanoporous metals, the elucidation of their basic plasmonic properties is crucial. The plasmonic properties of nanoporous metals can be tuned by using different strategies for their preparation (these compounds are obtained by synthetic routes) [88], including (i) templating, which permits a precise control of the size and structure of porous metallic structures, (ii) dealloying, which permits production of structures characterized by open nanopores, tunable pore sizes, structural properties and multifunctionality, and (iii) colloidal chemistry [6]. Very often nanoporous metallic nanoparticles are produced by using a combination of lithographic techniques (Section 3.2.1) and dealloying methods. An example is the synthesis of nanoporous gold nanoparticles, by depositing gold and silver on a substrate made of a silicon wafer or a glass slide [89,90].

On the other hand, a nanoporous silver structure was obtained by Yang and coworkers using a silver halide electroreduction process, which permitted getting a material with tunable pore size [91]. Nanoporous silver films of variable composition have been produced by Shen and O’Carroll using non-lithographic and heat-assisted methods [92]. 

Besides Au and Ag nanoparticles, aluminum has also been widely studied as a UV plasmonic material. In most cases, the preparation of suitable Al structures required several processing steps, ranging from chemical synthesis of Al nanoparticles to nanolithography for nanostructured films [93,94]. Garoli and co-workers have described the fabrication of nanoporous aluminum structures from Al_2_Mg_3_ alloy by means of a galvanic exchange reaction [95]. 

Jiang et al. have proposed the preparation of nanoporous Mg in a two-step process [96]. In the first step, Ti(Nb,Ta,V,Fe)_50_Cu_50_ alloys were melted in liquid Mg to synthesize interpenetrating phase composites. The Ti-rich phase was then etched by selective dissolution in 15 M aqueous HF solution for several minutes in an ultrasonic bath, followed by cleaning in deionized water and alcohol. On the other hand, Liu et al. synthesized nanoporous magnesium for hydrogen generation using physical vapor deposition, starting with Mg powders of large granulation [97].

### 3.3. Other Materials

**Figure 3:**
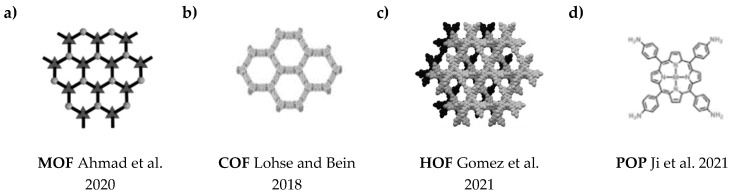
The structures of porous materials: (**a**) MOF [125], (**b**) COF [126], (**c**) HOF [127], (**d**) POP [110].

Recently, several new porous materials have been obtained for use in photocatalysis, including metal-organic frameworks (MOFs, Figure 3a), covalent organic frameworks (COFs, Figure 3b), hydrogen-bonded organic frameworks (HOFs, Figure 3c), and porous organic polymers (POPs, Figure 3d) [98,99,100,101]. MOFs composed of metal ions/clusters and organic linkers through coordination bonds, exhibit some unique features, which include periodic and well-defined structure, high specific surface area, structural diversity, and customizability. However, in addition to these advantages, they typically exhibit relatively low chemical stability and poor conductivity, which hinders their practical application [102,103]. COFs are a group of fully engineered crystalline materials obtained by polymerizing organic building blocks through strong covalent bonds [104,105,106,107]. HOFs consist of organic molecules linked through hydrogen bonding, exhibiting a specific structure and low density. They exhibit poor chemical stability, which also limits their application [108,109]. POPs are highly stable porous materials linked through strong covalent bonds based on organic molecules. Due to their undefined structure and irregular pores, it is difficult to gain adequate knowledge of the structure-activity relationship [110,111]. 

Of the aforementioned materials, COFs not only combine their advantages, but also offset their disadvantages, and as a result, they are attracting increasing scientific interest and are used in, e.g.,: gas adsorption and separation, detection, and catalysis [112,113,114,115,116]. 

The particularly desirable solid-state behavior of COFs makes them promising materials for photocatalytic applications [117,118,119]. In 2008. Wan et al. [120] described a boronic ester-based COF that exhibited solid-state behavior confirmed by a linear I-V profile. Stegbauer and co-workers published a paper in which hydrazone-bound COF was used for the first time as a photocatalyst for hydrogen evolution under visible light radiation [121]. The publication of these results has stimulated a rapid increase in the application of these materials in photocatalytic research, for example: in photocatalytic CO_2_ reduction, organic transformation, and in pollutant degradation [122,123,124].

## 4. Application of Nanomaterials as Photocatalysts 

Due to the use of solar radiation and other low-cost light sources, photocatalytic processes using photocatalysts show great application potential in four main areas: environmental, energy, biomedical, and chemical synthesis [128].

Heterogeneous photocatalysis is a relatively low-cost and sustainable technology for the treatment of many pollutants found in air and water, including organic compounds and heavy metals. Japan, the US, India, and China are major users of this technology, as evidenced in part by the number of research publications in the field. Like any method, it has both advantages and disadvantages. In general, the main advantages of conventional photocatalysts include chemical and physical stability, low cost, and environmental friendliness. The main limitations are the lack of solar sensitivity and lower efficiency, which encourages researchers to design strategies based on multiple photocatalysts to overcome the problems, such as recombination and low solar sensitivity, and further expand the processing capacity.

### 4.1. Photodegradation of Pollutants

Recently, increasing attention is paid to the search for safe and environmentally friendly methods of removing contaminants of organic compounds and inorganic compounds (these impurities are usually oxidized to simpler, less harmful compounds). Among others, photocatalytic degradation in the presence of suitable photocatalysts, often titanium(IV) oxide nanoparticles, is used to remove contaminants from the aqueous and gas phases. The first degradation of biphenyl and chlorobiphenyls in the presence of titanium(IV) oxide was performed by Carey and colleagues [129]. Since then, heterogeneous photocatalysis has gained importance as a potential method for the removal of environmental pollutants, such as the degradation of organic compounds [130], inorganic compounds [131], removal of odors from confined spaces [132], and destruction of bacteria in the presence of weak UV radiation [104].

Currently, the method of photodegradation in the presence of TiO_2_ and solar radiation is used to purify water by removing benzene, toluene, ethylbenzene, and xylene, among others [133,134]. 

**Figure 4:**
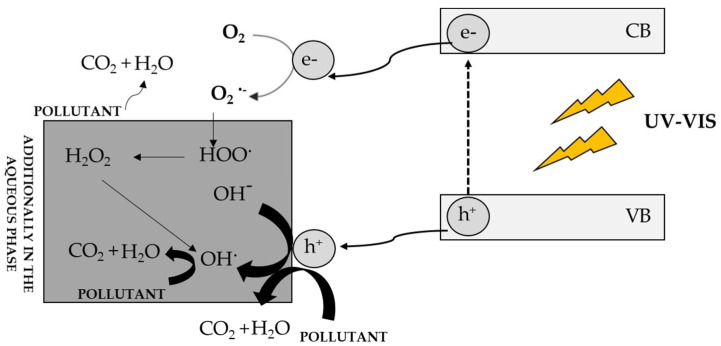
Mechanism of photocatalyst excitation and photodegradation of contaminants in the aqueous and gas phases [3].

Figure 4 shows the mechanism of semiconductor excitation and photodegradation of impurities [3]. As a result of the excitation of the semiconductor with a radiation quantum of the same or greater energy than the energy gap of the photocatalyst, an electron is excited from the VB band to the CB band. This leads to the formation of a reactive electron-hole pair, which reacts with environmental pollutants. The electron-hole exhibits oxidizing properties and can react directly with the contaminants present in the environment, while the electron reacts with molecular oxygen, generating a superoxide anion radical, which in aqueous environments is immediately converted to HO_2_ • and finally to do OH•. The resulting radicals rapidly oxidize impurities. If the process takes place in the aqueous phase, hydroxyl radicals can additionally be generated, which are also involved in the photodegradation of compounds. The final product of photodegradation is carbon dioxide and water, although other carbon-containing compounds at lower oxidation levels may be formed [135]. 

Examples of photocatalysts used to remove contaminants are:

- Nanoparticles: titanium(IV) oxide (removal of acetone [136], methyl blue, methyl orange [137]), SnO_2_ (removal of benzene [138]), ZnO (removal of methyl blue [139]; removal of methyl orange [140,141,142]), ZnS (removal of rhodamine [143]), Ag^0^ (removal of methyl blue [144]);
- Nanotubes: titanium(IV) oxide (removal of formaldehyde [145]), Ta_2_O_5_ (removal of toluene [146]);
- Nanocomposites: TiO_2_/CoxOy (phenol removal [147]);
- Nanowires: CdS (methyl orange removal [148]).

From among gas pollutants, nitrogen oxide and nitrogen dioxide are particularly toxic, the latter poses a particular threat to human health. Short-term exposure to a gas with a high concentration of NO_2_ leads to irritation of the upper respiratory tract, and long-term exposure can provoke chronic respiratory diseases, including cancer. Nitric oxide is several times less harmful to human health, but it oxidizes to nitrogen dioxide on contact with the air. One solution that can contribute to reducing the concentration of nitrogen oxides in the air is photocatalytic concrete. This material is the most widely used human construction material, so its use in the context of reducing the concentration of nitrogen oxides seems a promising idea. Embedded in the structure of photocatalytic concrete are molecules of a photocatalyst, which, as a result of absorption of solar radiation, initiate a chemical reaction or affect the rate of an already occurring reaction. The most commonly used photocatalyst in concrete technology is titanium (IV) oxide in the form of anatase [149].

### 4.2. Heterogeneous Photocatalysis in the Removal of Heavy Metals

As a result of heterogeneous photocatalysis, metals and metalloid elements are converted to less toxic (lower valence) forms and/or are permanently deposited on the semiconductor surface [150,151]. The reaction of photocatalytic removal of heavy metals from the aqueous phase begins with excitation of the semiconductor by absorption of energy equal to or exceeding the energy of its excited band. Doping of zinc oxide with selenium [152] and silver [153] enhances the photocatalytic performance of the nanocomposite and results in a significant reduction or complete removal of heavy metals, such as: Cd, Ni, Pb, Zn, Cu, Cr from the aqueous environment. Modification of titanium(IV) oxide surface with silica [154], graphene [155], organic acids [156], and sulfur compounds [157] also allows the elimination of heavy metals from the reaction mixture.

The metals present in trace amounts in the natural environment such as mercury (Hg), chromium (Cr), lead (Pb) and others are considered highly hazardous to human health. Environmental applications of heterogeneous photocatalysis include the removal of heavy metals such as (Hg), chromium (Cr), lead (Pb), cadmium (Cd), lead (Pb), arsenic (As), nickel (Ni), and copper (Cu). The photoreduction capability has also been used to recover expensive metals such as gold (Au), platinum (Pt), and silver (Au) from industrial wastewater [158].

### 4.3. CO_2_ Photoconversion and Water Photodecomposition

**Figure 5:**
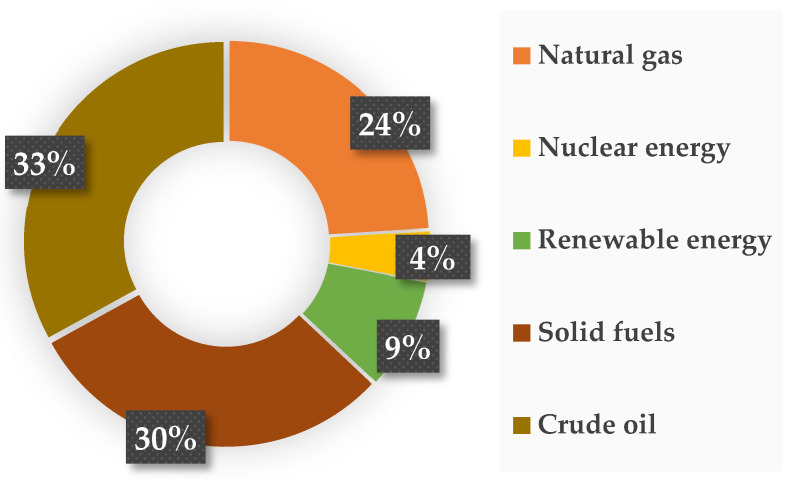
Main energy resources and their percentage contribution to energy production [161].

In addition to the above-mentioned applications, photocatalysts are now increasingly used in the transformation of carbon(IV) oxide to fuels or other compounds. All over the globe, there is an increasing demand for energy at an alarming rate, which is related to both continuous population growth and economic and technological development [159]. Figure 5 shows that more than 80% of the generated energy comes from burning fossil fuels, which include oil, natural gas, and solid fuels (mainly coal and lignite) [160]. 

Fossil fuels show great importance in the global economy, for this reason, they are referred to as strategic raw materials, however, their massive use around the world is associated with concerns about the sufficiency of energy for future generations [162]. At present, fossil fuel resources are heavily depleted [159]. Moreover, in addition to concerns about the sufficiency of fossil resources, attention has always been paid to their adverse environmental impact. The use of energy derived from the combustion of fossil fuels is associated with the emission of significant amounts of carbon(IV) oxide into the atmosphere, which in turn exacerbates the global warming problem [163]. According to forecasts, the energy demand will continue to increase, so it is necessary to find a solution that reconciles the ever-increasing demand for energy with the need to protect the environment [164]. The solution to this problem, in the main, is the development of energy from renewable resources, especially in the direction of obtaining alternative substitutes for transportation fuels. After all, one of the main directions of consumption of energy produced from fossil raw materials is the transportation sector, consuming as much as 60% of its total amount [164]. When obtaining an alternative energy source, the reduction of carbon(IV) oxide in the atmosphere should also be considered. Such an opportunity is provided by the photoconversion of carbon(IV) oxide to light hydrocarbons. Most photocatalysts in use reduce CO_2_ to carbon(II) oxide, methane, methanol, formic acid, or higher hydrocarbons. The energy required for this process is electromagnetic radiation of the desired wavelength. To date, this reaction has primarily used oxides and sulfides of transition metals, such as CdS [165], ZnIn_2_S_4_ [166], TiO_2_ [167], Bi_2_WO_6_ [168]. Yet, due to these materials’ low stability and lifetime, the search is constantly underway for a semiconductor material that both absorbs visible light and has an optimally located conduction band potential. The mechanism of photoconversion of carbon dioxide is based on the use of semiconductor materials to excite reactions involving solar radiation. The initiation of the redox reaction is a result of the photoexcitation of an electron (it is important that the refractory gap of the semiconductor material is smaller than the photon energy). The excited electrons are transferred from the valence band (VB) to the conduction band (CB). The generated charge carriers can move to the surface of the semiconductor material and react with substances adsorbed on the surface, such as CO_2_. Holes and electrons can recombine or combine in trap states. Table 2 shows examples of photocatalysts used in the photoconversion of carbon(IV) oxide reaction to hydrocarbons under UV radiation in the ultraviolet range and VIS radiation in the visible range.

One of the main alternative fuels that can replace existing fossil fuels is hydrogen. It is of great interest because of its characteristics; it is abundant, has a high heat of combustion, its combustion produces only water, and, most importantly, is environmentally friendly [178,179,180]. The element, in the presence of oxygen, burns with a nearly colorless, light blue flame with a relatively high propagation speed (2.7 m/s). The possibility of spontaneous ignition of a hydrogen-air mixture depends mainly on its concentration, for example, at 293 K the mixture can spontaneously ignite if the volume concentration of hydrogen is between 4 and 75%. Mixtures with particularly explosive properties are obtained in the hydrogen concentration range of 18–65%. Handling gaseous hydrogen is dangerous due to its flammability and explosiveness and requires extreme caution. At present, about 48% of hydrogen is generated by steam reforming of methane at elevated temperatures 30% is generated from oil, 18% from coal, and 4% from water electrolysis [181]. Among the best known methods for obtaining hydrogen is natural gas reforming [182]; coal or coke gasification [183], plasma technology [181], water electrolysis [183], photoelectrolysis [184], and biological methods [181]. This indicates that it can be extracted from a variety of feedstocks in many ways, although minimizing the cost of its production is currently a challenge for scientists and engineers. Currently, hydrogen is used as a fuel to power spacecraft, in fuel cells to provide heat and electricity, and to power some automobiles [185]. 

Photocatalytic reactions are the basis of technologies for obtaining a clean, environmentally safe energy carrier, which is hydrogen, not only from water but also from organic substances using the renewable energy source, which is the Sun. Photocatalytic hydrogen production is carried out in two ways: (i) photocatalytic splitting of water and (ii) photocatalytic reforming of organic compounds [186]. The first approach is based on the ability of water to reduce and oxidize by reacting with photogenerated electrons and positively charged “holes”, during the irradiation of semiconductors, in the presence of selected cocatalysts. The second approach is based on the ability of some organic species to donate electrons to the positive holes of the illuminated photocatalyst and be oxidized, generating proton ions, while photogenerated electrons reduce the latter to produce hydrogen in the presence of proper co-catalysts [187].

Table 3 shows examples of photocatalysts that can be used in the decomposition of water under the influence of UV radiation in the ultraviolet range and VIS radiation in the visible range.

## 5. Conclusions

This paper reviews the literature covering the synthesis and exemplary applications of nanomaterials as photocatalysts. These materials, metallic and semiconducting in nature, which can be excited by radiation in the UV-Vis range can potentially be used, among other applications, for the removal of pollutants from the environment (mainly on a laboratory scale). Over the years, research on the synthesis of photocatalysts has evolved considerably; from the use of transition metal oxides (e.g., TiO_2_/ZnO) to much more advanced materials. The research advances favor the development of heterogeneous photocatalysts, in which the absorption of light is shifted from the UV to the visible regime (sunlight) to utilize sunlight/white light for photocatalysis. The efficiency of the photocatalysis reaction depends on a number of factors, including: light absorption capacity/light intensity, the type of photocatalyst used, the concentration of a photocatalyst and contaminant particles, the pH of the reaction medium and others. Therefore, it is very important to determine the optimal amount of these factors for a given photocatalyst and type of pollutant. The process of photocatalysis has been studied for a long time on a laboratory scale, but unfortunately, its large-scale application is greatly hampered, for example, by blocking light penetration in thick coatings, leaching effect, difficulty in recovering the photocatalyst, etc. In addition, in natural systems, the rate of decomposition of pollutants is not limited by a time regime, unlike in industrial installations, where the technical challenge of photocatalytic processes, especially in heterogeneous systems, is unsatisfactory reaction kinetics.

Researchers’ interest in this topic is growing year by year, as evidenced by the steadily increasing number of papers in this thematic area. However, future work should focus on finding solutions for large-scale production, commercialization, and applications. Obtaining efficient, low-cost, stable, visible-light-activated photocatalysts continues to be a challenge in the field of photocatalysis. However, in my opinion, it is worthwhile to continue this work and to develop a highly efficient photocatalyst, which would be used, among other things, to obtain a pure energy carrier, such as hydrogen. Although photocatalytic reactions for obtaining hydrogen have been extensively studied, their overall efficiency towards hydrogen evolution is low. In many cases, satisfactory photocatalytic activity is only achieved when irradiated with UV light. For this reason, the search for new materials that are active and stable in the presence of sunlight is of great interest.

## Figures and Tables

**Table 1:** Comparison of the properties of traditional catalysts with photocatalysts [9].

Conventional Catalysts	Photocatalysts
- have a certain number of active centers with which the chemical reaction takes place and which, with the time of using the material, can be poisoned	- usually are semiconducting compounds (they can exist in a self-supporting form or can be deposited on a carrier), which can be excited by radiation from the UV-Vis range; for a semiconductor to photocatalyze a particular chemical reaction, the potential of such a process should be within the limits of the photocatalyst’s band gap, between the potential of the valence band and conduction
- formation of transition products as a result of the reaction of substrates with a catalyst	- generate reactive electron-hole pairs across the excited surface, which then interact with substrates
- modification of the course of the reaction, affecting the reduction of activation energy and increasing the rate of product formation	- do not react directly with reagents
- selective activity	- non-selective activity

**Table 2:** Examples of photocatalysts used in the photoconversion of carbon(IV) oxide reaction to hydrocarbons under UV radiation in the ultraviolet range and VIS radiation in the visible range.

Material	Type of Radiation	Ref.
TiO_2_ nanoparticles	UV-VIS	[169]
Ag/TiO_2_ nanoparticles	UV-VIS	[170]
Au/TiO_2_ nanoparticles	UV-VIS	[171]
I-TiO_2_ nanoparticles	UV	[172]
Co/Co_3_O_4_ nanoparticles	UV-VIS	[173]
AgBr/TiO_2_ nanocomposite	UV	[174]
ZnO/ZnTe nanocomposite	VIS	[175]
C-Na_2_TiO_3_ nanowires	UV-VIS	[176]
N- Na_2_TiO_3_ nanowires	UV-VIS	[177]
N- Na_2_TiO_6_ nanowires	UV-VIS	[177]

**Table 3:** Examples of photocatalysts that can be used in the decomposition of water under the influence of UV radiation in the ultraviolet range and VIS radiation in the visible range.

Material	Type of Radiation	Ref.
TiO_2_ nanoparticles	UV	[188]
Au/TiO_2_ nanoparticles	UV	[188]
Pt-TiO_2_ nanoparticles	UV	[189]
TiO_2_/F/Pt nanocomposite	UV	[190]
La:NaTaO_3_ nanocomposite	UV-VIS	[190]
RuO_2_/La-NaTaO_3_ nanocomposite	UV-VIS	[190]
SnO_2_ nanowires	VIS	[191]
SnO_2_/SnS_2_ nanowires	VIS	[191]

## Data Availability

Not applicable.
